# Constructing Directed Acyclic Graphs (DAGs) to Inform Tobacco Cessation Intervention Research: A Methodological Extension Using Evidence Synthesis

**DOI:** 10.3390/healthcare13222837

**Published:** 2025-11-08

**Authors:** Sanchita Sultana, Naiya Patel, Joseph Inungu

**Affiliations:** 1 Public Health Epidemiology, Washtenaw County Health Department, Ann Arbor, MI 48104, USA; sultanas@washtenaw.org; 2 EmpVic Research Public Health Solutions, Anand 388210, Gujarat, India; 3 School of Health Sciences, Central Michigan University, Mount Pleasant, MI 48859, USA; inung1j@cmich.edu

**Keywords:** tobacco cessation, directed acyclic graph, evidence synthesis, health disparities, socioeconomic factors, digital equity, provider engagement

## Abstract

Background: Tobacco use remains a leading preventable cause of morbidity and mortality in the United States, with persistent disparities in cessation outcomes across socioeconomic and racial groups. While numerous interventions exist, their effectiveness is shaped by complex interrelated factors at individual, social, and healthcare system levels. Identifying and modeling these causal pathways is essential to inform equitable intervention design. Methods: This study applied the Evidence Synthesis for Constructing Directed Acyclic Graphs (ESC-DAG) protocol to integrate empirical findings from 35 quantitative studies examining barriers and facilitators of tobacco cessation intervention uptake in the United States. Using the Andersen and Aday Health Services Research Model as a guiding framework, we extracted, harmonized, and synthesized significant causal relationships into a unified DAG, distinguishing exposures, outcomes, mediators, and confounders. Results: The integrated DAG revealed that structural factors such as socioeconomic disadvantage, digital inequities, rurality, and cultural barriers exerted substantial influence on cessation outcomes. These upstream determinants operated through mediators including motivation, treatment engagement, and access barriers, while healthcare system factors such as provider engagement and proactive outreach emerged as consistent facilitators. Digital access and culturally tailored interventions were identified as underexplored yet potentially high-impact pathways. Discussion: The ESC-DAG methodology provided a structured approach to visualize and synthesize causal mechanisms beyond traditional review synthesis, highlighting points of intervention at both policy and practice levels. The findings underscore the importance of multi-level strategies, including financial support, digital equity initiatives, provider outreach, and culturally tailored cessation services. Conclusions: By applying ESC-DAG methodology, this study contributes a novel causal framework for understanding disparities in tobacco cessation intervention uptake. The resulting DAG can inform future statistical modeling, simulation studies, and equity-focused program design, supporting more effective public health strategies to reduce smoking prevalence and associated inequities.

## 1. Introduction

Smoking is one of the leading preventable causes of premature death and health inequities in the United States. According to a study conducted in the U.S. from 2014 to 2019 using U.S. population data, a persistent sociodemographic inequality was identified. Although a similar or higher quit interest exists among non-White and lower socioeconomic status groups, significantly lower sustained cessation rates, i.e., only about 7.5% were observed. The tobacco cessation treatment uptake remained low, i.e., 34% and the disparities in treatment receipt did not improve significantly [1]. The NHIS data examination reported similar underlying trends in disparities in receiving professional cessation advice. Those who are older adults, reside in urban areas, have access to primary care, and are diagnosed with COPD were highly likely to receive tobacco cessation treatment assistance, disproportionately affecting rural, younger, uninsured, and racial minority smokers [2,3].

As per a recent CDC MMWR report, about 50% of adult smokers among 28.8 million U.S. adult smokers tried to quit, but only about 10% succeeded, and less than 40% utilized tobacco cessation interventions, i.e., medical or non-medical interventions [4]. Lower access to tobacco cessation interventions and lower sustained quitting are associated with lower socio-economic status and rural residence. Although the cessation intervention exists, access to pharmacotherapy, counselling, or other types of cessation interventions remains limited due to unaligned care coordination, cost, or lower healthcare engagement [2,3,5]. Prior work has identified key barriers and facilitators of cessation intervention utilization in the United States at both the population and healthcare system levels. The key barriers identified across diverse cessation intervention types are digital inequities for digital interventions, socioeconomic disadvantage, and low motivation at the population level. At the healthcare system level, barriers to care access and inadequate healthcare provider engagement have been identified as further system-level barriers in effective tobacco cessation intervention utilization in the U.S., while the key facilitators identified are financial incentives, culturally tailored interventions, and digital engagement strategies [6].

The effectiveness of treatment interventions outside a strict clinical trial environment is affected by several confounding barriers and facilitating factors [7]. It is crucial to identify such factors to develop a Directed Acyclic Graph (DAG), a visual representation of complex relationships between key factors affecting the exposure and outcome variables relationship in any observational data study, as it informs the statistical modeling for causal inferences [6,7,8]. A conventional statistical model due to parametric assumptions might not capture the comprehensive causal relationship of a study context; however, a DAG can graphically depict the complex causal relationship between a key exposure and outcome factors. According to PubMed, Embase, Web of Science, and Google searches, there exists no scientific literature that develops evidence-based DAG on factors affecting tobacco cessation intervention utilization in the United States. As established, it is vital to build such DAGs to identify causal relationships that affect the tobacco cessation intervention utilization to improve the health inequities and smoking prevalence rate in the United States. Although evidence-based cessation intervention exists, there is a need to identify measurable factors that affect the causal relationship between socioeconomic factors and cessation or quit outcomes in the presence of key confounders. Hence, this study builds upon a prior systematic review to develop an evidence synthesis DAG [6].

## 2. Materials and Methods

### 2.1. Study Design and Evidence Base

This study utilized the Evidence Synthesis for Constructing Directed Acyclic Graphs (ESC-DAG) protocol to identify the causal factors affecting tobacco cessation intervention uptake in the United States. In observational research, the ESC-DAG methodology helps to integrate causal relationships from existing empirical evidence [9].

A total of 35 studies were identified in the prior extension of this work, a systematic review [6] about barriers and facilitators to tobacco cessation interventions at the population and healthcare system levels. The study included randomized control trials, quasi-experimental, and observational quantitative studies that examined determinants of smoking cessation intervention uptake and treatment disparities at both the population and healthcare system level.

Although ESC-DAG methodology typically integrates multiple evidence sources, this study used a prior systematic review that synthesized 35 quantitative studies examining barriers and facilitators of tobacco cessation intervention uptake in the United States. Given the methodological rigor, comprehensive scope, and quality appraisal within that review, it served as a consolidated and sufficient evidence base for DAG construction consistent with established ESC-DAG applications.

### 2.2. ESC-DAG Protocol Application

The mapping stage included edge identification and coding in DAGitty software (v 3.1) [10]. Each study included was reviewed to extract empirically supported causal relationships/arrows (interchangeably used with terminology edges) between exposure and outcome variables. Edges that were statistically significant between exposure and outcome variables and coded within the original study context were extracted verbatim. The direct and indirect pathways were established without any directionality rules implied to preserve the original study context.

The translation stage included thematic grouping and categorization by imposing ESC-DAG protocol directionality rules. The conceptual framework used to determine the temporality and directionality of constructs was frequently the Andersen and Aday Health Services Research framework [11]. Overlapping constructs, such as digital access and digital inequities, were combined into a single standardized category. The bidirectionality was assessed, and constructs were aligned with theoretical determinants from the conceptual framework.

The integration stage involved synthesis into a final compiled ESC-DAG. The harmonized constructs and their edges were synthesized into a single DAG that included confounders, mediators, and potentially other effect modifiers. Two reviewers (N.P. and S.S.) independently conducted edge extraction from each included study, identifying statistically supported directional relationships (edges) between constructs. The third reviewer (J.I.) synthesized the independent extraction files and conducted a consensus reconciliation. All reviewers jointly applied thematic grouping and temporal alignment following the Andersen and Aday Health Services Research Model. N.P. implemented the final harmonized Directed Acyclic Graph (DAG) using DAGitty software, and all three reviewers validated node placement and directionality through iterative review.

### 2.3. Conceptual Framework and Quality Assurance

The Andersen and Aday Health Services Research Model was utilized as an organizing framework to contextualize constructs across predisposing, enabling, and need-based factors. The protocol recognized the need for a conceptual framework to inform its translation stage, ensuring that the DAG represented statistical associations aligned with established theory on healthcare access and utilization.

## 3. Results

### 3.1. Mapping Stage Findings

A total of 35 included studies identified a wide range of causal pathways that linked individual, social, and structural factors to smoking cessation outcomes (Table 1). Each study contributed multiple causal pathways, i.e., edges establishing several potential pathways. Frequently mapped factors included socioeconomic disadvantage, digital access and literacy, motivation to quit, and healthcare system barriers, i.e., provider engagement and treatment availability (Table 2). Nicotine Replacement Therapy (NRT), financial incentives, and mobile-based supports were recurrent intervention-specific nodes. As depicted in Table 1, the yellow nodes represent outcome variables, the blue nodes represent exposure variables, the green nodes represent mediators, and the grey nodes represent confounders, as identified across each included study.

Across the 35 included studies, socioeconomic disadvantage was the most frequently identified barrier (reported in 54% of studies), followed by digital inequities or low digital access (43%), low motivation or readiness to quit (37%), and healthcare access barriers (34%). The most frequently reported facilitators were provider engagement or proactive outreach (49%), financial incentives (40%), digital engagement supports (34%), and culturally tailored interventions (29%). These constructs were consistently represented across RCTs, quasi-experimental, and observational studies, indicating their robustness within the synthesized evidence base.

### 3.2. Translation Stage and Integration of Extracted DAGs

Following the extraction of individual Directed Acyclic Graphs (DAGs), a structured translation procedure was implemented to harmonize constructs and establish temporal consistency across studies (Table A1 and Table A2). This process comprised three sequential steps:

#### 3.2.1. Semantic Aggregation

Extracted nodes and edges, i.e., arrows between the nodes (hereafter referred to as edges or causal pathways), were systematically reviewed to identify and reconcile conceptual overlaps. Constructs with equivalent or closely related meanings (e.g., “technology literacy barriers” and “digital access challenges”) were merged into unified categories such as “Digital Access Barriers” or “quitting/vaping/smoking/cessation”. Similarly, outcome constructs, including quit attempt, abstinence, and relapse prevention, were standardized under the category Smoking Cessation. This ensured terminological coherence and reduced redundancy across datasets.

#### 3.2.2. Temporal Re-Alignment

Constructs were organized according to their logical order of influence into upstream determinants, interventions, mechanisms, behaviors, and outcomes. Temporal sequencing was inferred using established causal reasoning and a health behavior framework, including Andersen and Aday’s Behavioral Model of Health Services Use. For example, distress consistently preceded intervention access and was thus retained as an upstream determinant rather than reclassified as a mechanism.

#### 3.2.3. Directional Mapping and Validity Assessment

Causal edges were redrawn only when directionality was explicitly supported by primary data or demonstrated consistency across multiple sources. Recourse theory and temporal precedence informed the polarity of edges, thereby avoiding ambiguous or circular paths. Constructs with uncertain directionality were excluded. Face validity of the integrated map was assessed iteratively through expert consensus (*n* = 3) and triangulated with an established conceptual model.

No single study design was privileged. Instead, causal edges were extracted based on statistical support and mapped uniformly across RCTs, quasi-experimental, and observational studies. Edge direction and placement within the DAG were confirmed using cross-study consistency, temporal precedence, and theoretical plausibility.

### 3.3. Integrated DAG

**Figure 1:**
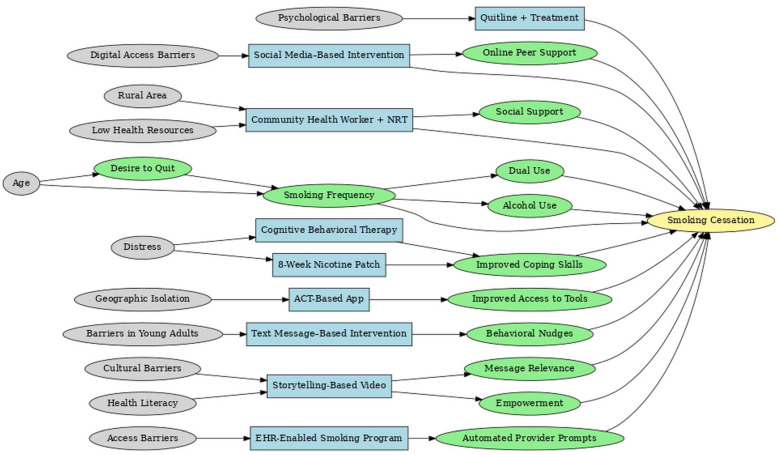
Integrated DAG Establishing Causal Pathways from Exposure to Outcome Nodes.

The final Integrated Directed Acyclic Graph (iDAG) presents a comprehensive systems-level representation of the causal architecture underpinning smoking cessation interventions (Figure 1). By synthesizing 35 individual DAGs into a single unified framework, the iDAG illustrates the multilevel and interdependent processes that characterize tobacco cessation through the lens of Andersen and Aday’s Health Services Research conceptual model framework. It delineates how structural inequities cascade through access barriers, intervention modalities, psychological mechanisms, and behavioral mediators to shape cessation outcomes ultimately.

#### 3.3.1. Structural: Upstream Determinants

The leftmost domain of the IDAG comprises upstream structural determinants, encompassing demographic and contextual factors such as psychological distress, age, digital access limitations, rural residence, health literacy, and psychosocial barriers. Although these constructs are largely non-manipulable, they are foundational in conditioning access to interventions and moderating treatment effectiveness. For example, psychological distress initiates two distinct causal trajectories: one leading toward Cognitive Behavioral Therapy (CBT) and another toward Nicotine Replacement Therapy (NRT) sampling, both mediated by improvements in coping capacity.

#### 3.3.2. Intervention: Modality-Specific Pathways

The intervention layer identifies modality-specific pathways that align with the aforementioned upstream constraints. Geographic isolation directs individuals toward Acceptance and Commitment Therapy (ACT) based mobile applications, facilitating access to evidence-based tools. Digital access limitations and low health literacy channel participants toward social media and storytelling-based interventions that enhance message relevance and peer engagement. Psychological barriers are associated with the use of quitline services, which mitigate in-person access limitations. Each intervention node is linked to distinct mechanisms of action, highlighting how delivery modality and contextual appropriateness influence downstream behavioral change.

#### 3.3.3. Mechanistic: Mediators of Change

Centrally positioned within the iDAG are mechanistic nodes such as improved coping skills, empowerment, social support, and behavioral nudges that mediate the causal effects of interventions. Their centrality underscores their critical role in translating intervention exposure into behavioral outcomes. CBT, NRT, and ACT-based approaches all converge on coping skill enhancement, suggesting strong theoretical coherence across modalities. Mechanisms such as automated provider prompts in electronic health record (EHR) systems and online peer engagement in digital platforms are intervention-specific, reinforcing the importance of causal pathway specificity in understanding intervention efficacy.

#### 3.3.4. Behavioral: Modifiable Risk Factors

The behavioral layer comprises dynamic, modifiable mediators, including smoking frequency, dual use (cigarettes and vaping), alcohol consumption, and motivation to quit. These behavioral constructs link psychological mechanisms to cessation outcomes and capture known confounding relationships. For example, dual use and alcohol consumption often co-occur and collectively diminish cessation likelihood. Age and motivation to quit further moderate these dynamics, highlighting the complex feedback between readiness to change, behavioral risk factors, and cessation success.

#### 3.3.5. Outcome: Smoking Cessation

At the terminal point of the IDAG lies the smoking cessation node, where all causal pathways converge. Cessation outcomes are influenced by both direct intervention effects and indirect pathways mediated through behavioral and psychological mechanisms. Notably, no upstream determinant connects directly to cessation without traversing intermediate nodes, underscoring the mediated and multistage nature of effective tobacco control strategies.

## 4. Discussion

The integrated ESC-DAG establishes complex and multifactorial causal pathways that affect tobacco cessation intervention outcomes in the United States. One of the key identified barriers was structural, i.e., socioeconomic disadvantage, digital inequities, and rurality, which affected access to interventions. These factors affected both the direct cessation outcomes and indirect mediators, including motivation, engagement, and treatment adherence. One of the key identified facilitators was provider engagement, emphasizing that healthcare system interactions remain crucial for cessation success.

The findings of this study are consistent with emerging international evidence emphasizing the importance of cultural tailoring and digital innovation in smoking cessation interventions. For instance, Bhatt et al. (2024) demonstrated that culturally and disease-specific multi-component interventions significantly improved cessation rates among patients attending non-communicable disease clinics in India, supporting the present study’s identification of cultural adaptation as a key facilitator [45]. Similarly, Okpako et al. (2024) found substantial interest in digital and virtual-reality–based cessation supports among smokers in Great Britain, reinforcing our DAG-derived conclusion that digital access and engagement pathways are pivotal to enhancing intervention reach and adherence [46].

Some of the underexplored pathways, i.e., cultural tailoring, minority stress, and stigma, emphasize crucial gaps in existing evidence as only a few edges supported these domains from existing empirical studies. This study has several limitations. First, the evidence base was restricted to studies conducted in the United States, which may limit the generalizability of the resulting DAG to other healthcare or cultural contexts. Second, as the synthesis relied on published quantitative studies, potential publication bias cannot be fully excluded, despite the comprehensive search and inclusion strategy of the source review. Third, challenges arose in harmonizing conceptually overlapping constructs such as stress, depression, and minority stress. While constructs were consolidated using theoretical and empirical alignment, this harmonization may have reduced specificity in some psychosocial pathways.

### 4.1. Methodological Contributions

This study demonstrates the added value of the ESC-DAG methodology over traditional narrative or systematic reviews. By translating empirical evidence into a unified graphical model, we identified confounders, mediators, and moderators that are often overlooked in conventional statistical synthesis. Unlike meta-analysis, which emphasizes effect sizes, ESC-DAG enables researchers to map relationships across diverse study designs and uncover the underlying causal architecture. This methodological innovation is particularly valuable for complex behavioral and health services interventions, where context and interaction effects are critical.

### 4.2. Policy and Practice Implications

The integrated DAG provides insights for designing equity-focused and targeted cessation interventions. It encompasses digital equity, socio-economic support, provider engagement, and cultural tailoring, which reinforces the need for multi-level interventions addressing not only individual motivation but also other systemic barriers to healthcare access.

The integrated DAG could be utilized to guide confounder adjustment in observational studies of tobacco cessation interventions by applying DAG-informed simulation models to predict the potential impact of scaling specific strategies. It could guide the design of tailored digital platforms that account for motivation levels, literacy, and socio-economic context. Beyond visualizing causal relationships, the integrated ESC-DAG can serve as a strategic framework to prioritize interventions and resource allocation. For instance, structural determinants such as digital inequities and socioeconomic disadvantage occupy upstream positions in the DAG, influencing multiple downstream mediators including motivation, engagement, and access to cessation support. Addressing these upstream nodes through digital equity initiatives (e.g., subsidized internet access, device provision, digital literacy training) or financial support mechanisms (e.g., free NRT, incentive-based programs) is likely to yield broader system-level impact than focusing solely on individual-level behavior change. Conversely, downstream facilitators such as provider engagement and motivational support can be targeted for shorter-term implementation or pilot interventions.

The DAG also provides a foundation for future simulation modeling and quasi-experimental trials by identifying high-priority mediators for targeted testing. For example, a comparative trial could evaluate whether addressing digital inequities produces greater cessation gains than financial incentive programs within resource-constrained settings. Furthermore, by mapping interdependencies between structural and psychosocial factors, the DAG can guide the sequencing of interventions, informing whether structural interventions should precede or accompany behavioral supports.

## 5. Conclusions

This study extends the methodological application of ESC-DAG to tobacco cessation research, synthesizing evidence from 35 studies into a unified causal framework. The integrated DAG highlights the central role of structural inequities, digital access, and provider engagement in shaping cessation outcomes.

By visualizing these relationships, our analysis demonstrates how DAGs can inform statistical modeling, guide intervention design, and prioritize equity-oriented policy solutions. Future research should build on this framework through simulation studies, quasi-experimental evaluations, and longitudinal designs to test targeted strategies for reducing disparities in cessation uptake.

## Figures and Tables

**Table 1:** Mapping stage, individual study implied DAGs.

Study	Graph
[12]	** 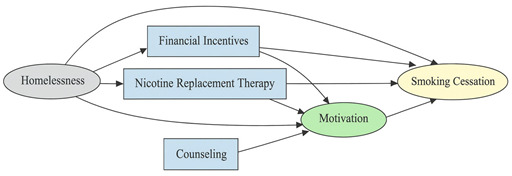 **
[13]	** 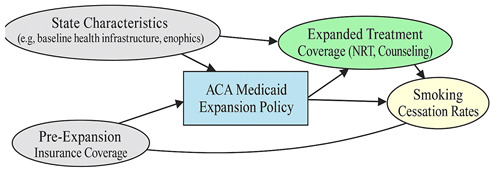 **
[14]	** 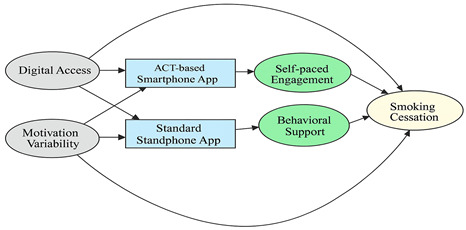 **
[15]	** 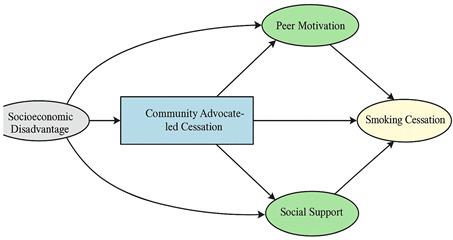 **
[16]	** 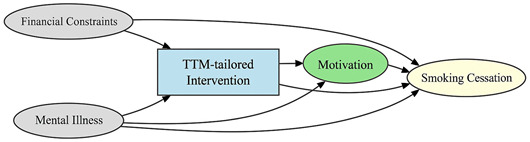 **
[17]	** 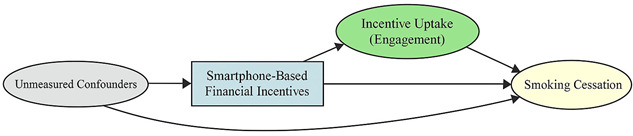 **
[18]	** 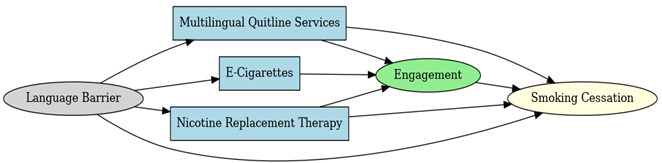 **
[19]	** 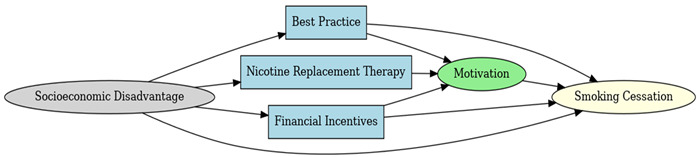 **
[20]	** 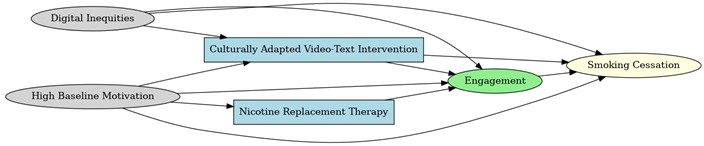 **
[21]	** 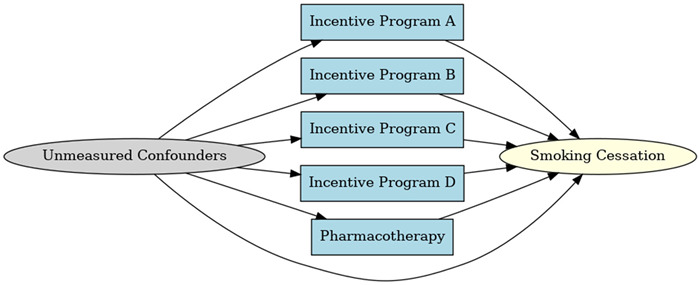 **
[22]	** 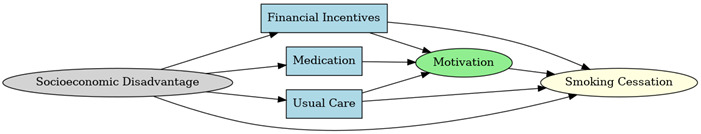 **
[23]	** 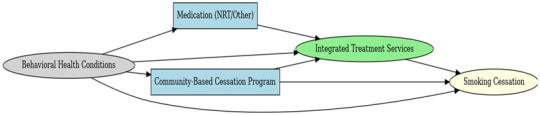 **
[24]	** 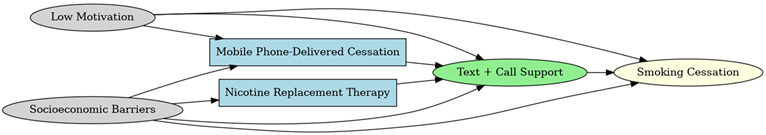 **
[25]	** 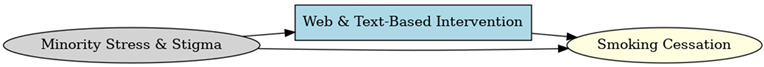 **
[26]	** 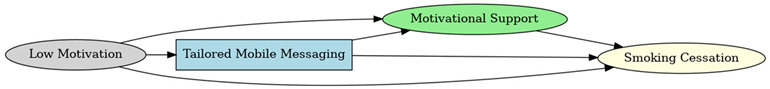 **
[27]	** 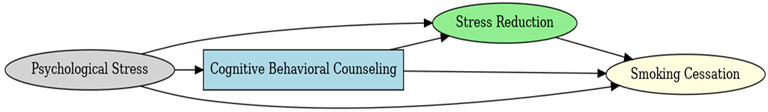 **
[28]	** 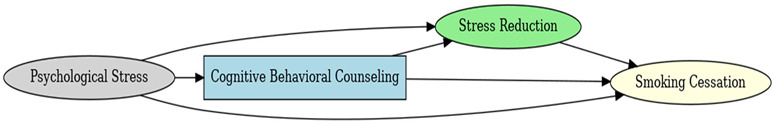 **
[29]	** 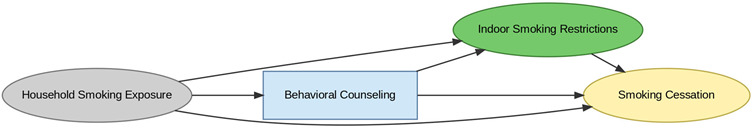 **
[30]	** 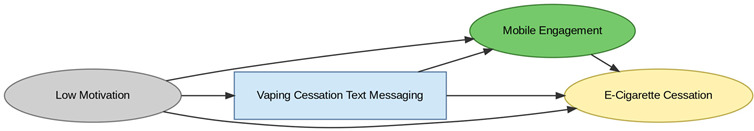 **
[31]	** 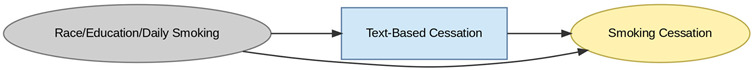 **
[32]	** 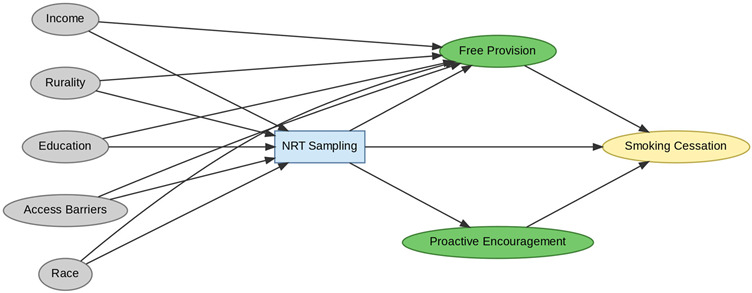 **
[33]	** 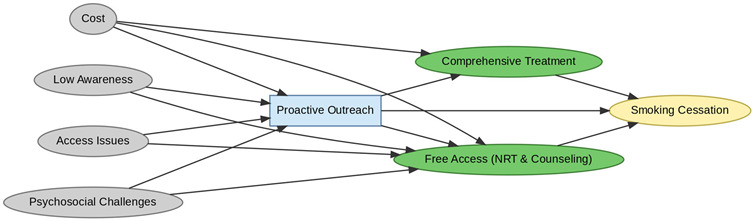 **
[34]	** 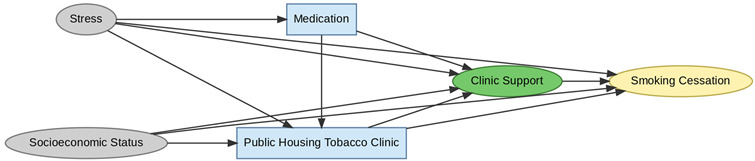 **
[35]	** 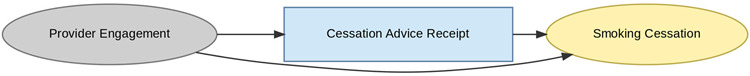 **
[36]	** 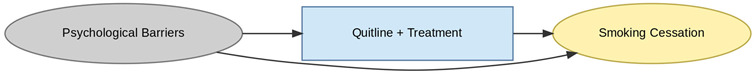 **
[37]	** 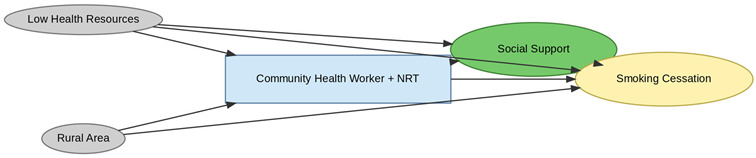 **
[38]	** 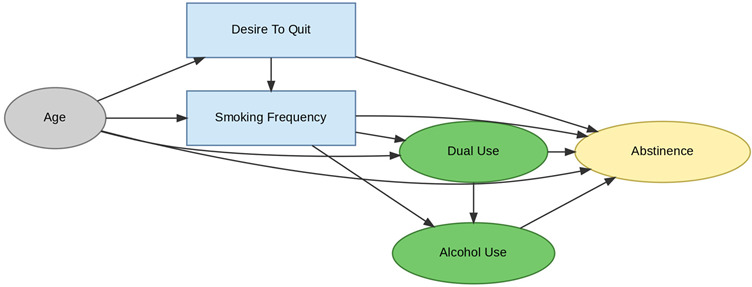 **
[39]	** 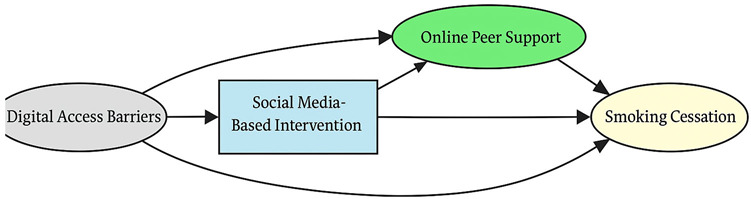 **
[40]	** 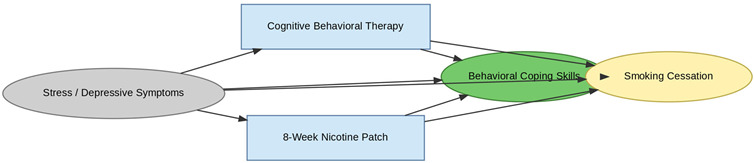 **
[41]	** 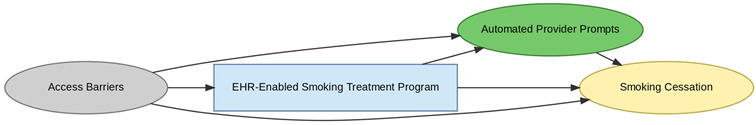 **
[42]	** 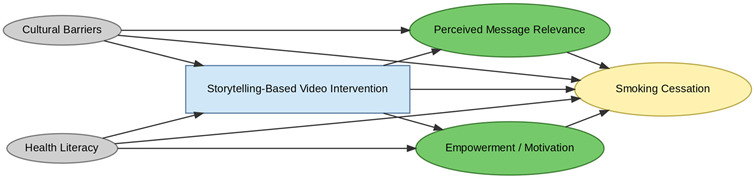 **
[43]	** 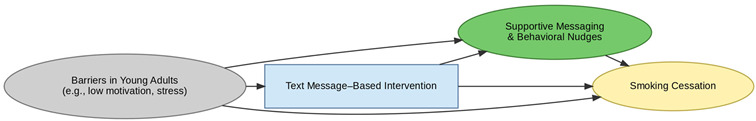 **
[44]	** 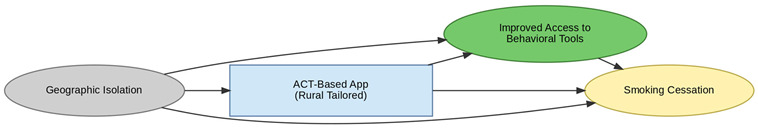 **

**Table 2:** Most frequently and consistently identified barriers and facilitators.

Category	Top Constructs Identified	Frequency (Number of Studies)	Illustrative Pathways
**Barriers**	1. Socioeconomic disadvantage	19	Income → Access barriers → Cessation outcome
	2. Digital inequities/low digital literacy	15	Digital access → Engagement → Cessation outcome
	3. Low motivation/readiness to quit	13	Motivation → Engagement → Cessation outcome
	4. Rurality/geographic isolation	10	Rural residence → Access barriers → Cessation outcome
	5. Psychological stress/depression	9	Stress → Adherence → Cessation outcome
**Facilitators**	1. Provider engagement/proactive outreach	17	Provider engagement → Cessation advice → Cessation outcome
	2. Financial incentives	14	Financial support → Treatment adherence → Cessation outcome
	3. Digital engagement/mobile-based support	12	Digital access → Self-paced engagement → Cessation outcome
	4. Culturally tailored interventions	10	Cultural tailoring → Motivation → Cessation outcome
	5. Social support/peer encouragement	8	Peer support → Motivation → Cessation outcome

## Data Availability

No new data was generated.

## Appendix A

**Table A1:** Translation stage results of included studies.

Edge	Temporality	Face Validity	Recourse to Theory	Final Action	Pathway Type
**Age → Desire to Quit**	Valid	Plausible	Supported in behavioral models	Retain	Confounder
**Age → Smoking Frequency**	Valid	Valid	Risk exposure literature supports	Retain	Confounder
**Age → Dual Use**	Valid	Supported by age-based substance use research	Yes	Retain	Confounder
**Smoking Frequency → Dual Use**	Valid	Strong behavioral linkage	Supported	Retain	Mediator
**Desire to Quit → Smoking Frequency**	Valid	Valid	TTM & quit models	Retain	Mediator
**Dual Use → Alcohol Use**	Valid	Comorbid use theory	Strong support	Retain	Mediator
**Dual Use → Smoking Cessation**	Valid	Plausible	Mixed evidence	Retain (flag for sensitivity)	Mediator
**Alcohol Use → Smoking Cessation**	Valid	Mixed support	Substance relapse theory	Retain	Mediator
**Cognitive Behavioral Therapy → Improved Coping**	Valid	Strong support	Core of CBT	Retain	Mediator
**Improved Coping Skills → Smoking Cessation**	Valid	Plausible	Strong in addiction theory	Retain	Mediator
**Distress → Cognitive Behavioral Therapy**	Valid	Yes	Routine clinical pathway	Retain	Confounder
**Distress → 8-Week Nicotine Patch**	Valid	Common clinical response	Yes	Retain	Confounder
**Distress → Improved Coping Skills**	Valid	Established stress models	Yes	Retain	Confounder
**Geographic Isolation → ACT-Based App**	Valid	Yes	Rural access models	Retain	Confounder
**ACT-Based App → Improved Access to Tools**	Valid	Strong	Valid	Retain	Mediator
**Improved Access to Tools → Smoking Cessation**	Valid	Behavioral support theory	Yes	Retain	Mediator
**Barriers in Young Adults → Text Msg Intervention**	Valid	Known access issue	Valid	Retain	Confounder
**Text Msg Intervention → Behavioral Nudges**	Valid	Behaviorism support	Yes	Retain	Mediator
**Behavioral Nudges → Smoking Cessation**	Valid	Supported	Yes	Retain	Mediator
**Cultural Barriers → Storytelling Video**	Valid	Strong support	Yes	Retain	Confounder
**Health Literacy → Storytelling Video**	Valid	Supported	Yes	Retain	Confounder
**Storytelling Video → Message Relevance**	Valid	Yes	Yes	Retain	Mediator
**Message Relevance → Smoking Cessation**	Valid	Plausible	Yes	Retain	Mediator
**Health Literacy → Empowerment**	Valid	Supported	Yes	Retain	Mediator
**Empowerment → Smoking Cessation**	Valid	Supported	Yes	Retain	Mediator
**Access Barriers → EHR Intervention**	Valid	Structural logic	Yes	Retain	Confounder
**EHR Intervention → Provider Prompts**	Valid	Plausible	Systematic evidence	Retain	Mediator
**Provider Prompts → Smoking Cessation**	Valid	Workflow behavior support	Yes	Retain	Mediator
**Digital Access Barriers → Social Media Intervention**	Valid	Digital divide evidence	Yes	Retain	Confounder
**Social Media Intervention → Online Peer Support**	Valid	Plausible	Yes	Retain	Mediator
**Online Peer Support → Smoking Cessation**	Valid	Peer-based models	Yes	Retain	Mediator
**Rural Area → CHW + NRT**	Valid	Intervention targeting	Yes	Retain	Confounder
**Low Health Resources → CHW + NRT**	Valid	Targeting underserved	Yes	Retain	Confounder
**CHW + NRT → Social Support**	Valid	Social pathway logic	Yes	Retain	Mediator
**Social Support → Smoking Cessation**	Valid	Strong evidence	Yes	Retain	Mediator
**Psychological Barriers → Quitline + Treatment**	Valid	Valid	Yes	Retain	Confounder
**Quitline + Treatment → Smoking Cessation**	Valid	Supported	Strong theoretical support	Retain	Mediator

**Table A2:** Operational definition of key constructs harmonized in the iDAG.

Harmonized Construct	Operational Definition	Distinct From	Source Constructs
**Psychological Distress**	Emotional or mental strain interfering with readiness to quit or treatment engagement	Minority Stress, Depression	“mental health”, “stress”, “emotional burden”
**Minority Stress**	Stressors from identity-based discrimination, stigma, or exclusion	General Stress, Socioeconomic Disadvantage	“racial stigma”, “LGBTQ discrimination”, “social bias”
**Digital Access Barriers**	Limited access to or usability of digital cessation tools due to device, connectivity, or literacy gaps	Health Literacy, Technological Engagement	“no smartphone”, “low digital literacy”, “poor access”
**Socioeconomic Disadvantage**	Structural deprivation limiting intervention access or adherence	Minority Stress, Rural Residence	“low income”, “unemployment”, “housing instability”
**Provider Engagement**	Direct action by healthcare providers to recommend, facilitate, or reinforce cessation	Passive Access, System Navigation	“counseling”, “EHR trigger”, “referral to services”
**Coping Skills Enhancement**	Mechanisms that strengthen emotional self-regulation and relapse prevention	Empowerment, Support	“stress management”, “resilience training”, “CBT”
**Motivation to Quit**	Individual’s readiness and intention to initiate cessation	Self-Efficacy, Knowledge	“desire to quit”, “stage of change”, “quit intention”
**Social Support**	External encouragement or assistance from family, peers, or community	Provider Engagement, Empowerment	“family support”, “peer encouragement”, “group sessions”
**Mobile App Interventions**	Use of structured, evidence-based cessation content delivered via smartphone apps	Quitlines, Storytelling	“ACT app”, “digital CBT”, “motivational text”
**Financial Incentives**	Monetary or material rewards linked to cessation behavior	Social Support, Intrinsic Motivation	“cash rewards”, “gift cards for abstinence”
